# Detection of relevant extracardiac findings on coronary computed tomography angiography vs. invasive coronary angiography

**DOI:** 10.1007/s00330-021-07967-x

**Published:** 2021-06-15

**Authors:** Dominik Laskowski, Sarah Feger, Maria Bosserdt, Elke Zimmermann, Mahmoud Mohamed, Benjamin Kendziora, Matthias Rief, Henryk Dreger, Melanie Estrella, Marc Dewey

**Affiliations:** 1 Department of Radiology, Charité – Universitätsmedizin Berlin, Charitéplatz 1, 10117 Berlin, Germany; 2 Department of Cardiology, Charité – Universitätsmedizin Berlin, Charitéplatz 1, 10117 Berlin, Germany; 3 Berlin Institute of Health and DZHK (German Centre for Cardiovascular Research) Partner Site, Berlin, Germany

**Keywords:** Computed tomography angiography, Coronary artery disease, Coronary angiography, Chest pain, Incidental findings

## Abstract

**Objectives:**

To compare the detection of relevant extracardiac findings (ECFs) on coronary computed tomography angiography (CTA) and invasive coronary angiography (ICA) and evaluate the potential clinical benefit of their detection.

**Methods:**

This is the prespecified subanalysis of ECFs in patients presenting with a clinical indication for ICA based on atypical angina and suspected coronary artery disease (CAD) included in the prospective single-center randomized controlled Coronary Artery Disease Management (CAD-Man) study. ECFs requiring immediate therapy and/or further workup including additional imaging were defined as clinically relevant. We evaluated the scope of ECFs in 329 patients and analyzed the potential clinical benefit of their detection.

**Results:**

ECFs were detected in 107 of 329 patients (32.5%; CTA: 101/167, 60.5%; ICA: 6/162, 3.7%; *p* < .001). Fifty-nine patients had clinically relevant ECFs (17.9%; CTA: 55/167, 32.9%; ICA: 4/162, 2.5%; *p* < .001). In the CTA group, ECFs potentially explained atypical chest pain in 13 of 101 patients with ECFs (12.9%). After initiation of therapy, chest pain improved in 4 (4.0%) and resolved in 7 patients (6.9%). Follow-up imaging was recommended in 33 (10.0%; CTA: 30/167, 18.0%; ICA: 3/162, 1.9%) and additional clinic consultation in 26 patients (7.9%; CTA: 25/167, 15.0%; ICA: 1/162, 0.6%). Malignancy was newly diagnosed in one patient (0.3%; CTA: 1/167, 0.6%; ICA: 0).

**Conclusions:**

In this randomized study, CTA but not ICA detected clinically relevant ECFs that may point to possible other causes of chest pain in patients without CAD. Thus, CTA might preclude the need for ICA in those patients.

**Trial registration:**

NCT Unique ID: **00844220**

**Key Points:**

*• CTA detects ten times more clinically relevant ECFs than ICA.*

*• Actionable clinically relevant ECFs affect patient management and therapy and may thus improve chest pain.*

*• Detection of ECFs explaining chest pain on CTA might preclude the need for performing ICA.*

**Supplementary Information:**

The online version contains supplementary material available at 10.1007/s00330-021-07967-x.

## Introduction

Coronary computed tomography angiography (CTA) and invasive coronary angiography (ICA) are both well-established methods for the assessment of cardiac and coronary anatomy and pathology. However, CTA is a radiological, noninvasive technique whereas ICA is a cardiological, invasive method. While both CTA and ICA also visualize surrounding structures, such as lungs, mediastinum, and chest wall, CTA is clearly superior to ICA in this respect. Detailed evaluation of adjacent anatomy allows identification of extracardiac findings (ECFs) which may potentially explain the patient’s symptoms or require further workup and therapy. This may be especially important for patients in whom cardiac pathologies have been ruled out, but chest pain still persists. Furthermore, patients in whom CTA rules out CAD while simultaneously detecting ECFs explaining chest pain might be spared an ICA. Previous studies show that ECFs are common in patients undergoing CTA [1–18]. On the other hand, there is an ongoing debate in the scientific community on the need to look for ECFs in CTA scans [19, 20]. At the same time, results on the clinical relevance of ECFs vary strongly from one study to the next, whereas robust evidence regarding the potential clinical benefit of detecting such findings with long-term follow-up is virtually not existing. We found only one study on the follow-up assessment of ECFs in patients with chest pain. In this study, some of the ECFs were identified as treatable causes of the patients’ chest pain [2].

The Coronary Artery Disease Management (CAD-Man) prospective randomized controlled trial has already shown that CTA performed instead of ICA in patients with atypical angina and a low to intermediate CAD risk significantly enhances the diagnostic yield of coronary angiography, reduces minor procedural complications, and shortens hospitalization compared with direct ICA [21]. The primary aim of this substudy was to analyze the potential clinical benefit of the identification of clinically relevant ECFs on CTA and ICA in patients presenting with atypical chest pain by assessing the scope of recommended diagnostic procedures and therapeutic consequences following the detection of such ECFs. The ancillary aim of our substudy was to investigate the impact of patient age, sex, and smoking behavior on the prevalence of detected ECFs.

## Materials and methods

### Study design and participants

We performed a subanalysis of ECFs based on data from the prospective single-center randomized controlled Coronary Artery Disease Management (CAD-Man) study, in which the protocol included ECFs as a prospectively collected item with clear management recommendations to patients based on predefined findings [21]. Recruitment of study participants has been described in detail elsewhere [21]. Briefly, between February 18, 2009, and August 27, 2015, we enrolled 340 patients with atypical angina or chest pain referred for ICA due to suspected CAD. The patients were randomly assigned to CTA (168/340) and ICA (172/340). Atypical angina was defined as the appearance of no more than two of the three criteria for typical angina pectoris, which are retrosternal chest pain, precipitation of pain by exertion, and rapid relief of symptoms at rest or within 30 s to 10 min following nitroglycerine administration [22].

### CT and ICA procedure

CT examinations were performed preferably in the early morning when individuals have lower heart rates. A contrast agent with an iodine concentration of 350 mg/ml (Iobitridol, Xenetix® 350, Guerbet) was used. Patients in the ICA group underwent diagnostic testing after hospital admission according to clinical practice at our institution, which follows European guidelines [23]. The same contrast agent as for CTA was used. ICA examinations were evaluated independently by at least two board-certified cardiologists with a minimum of 5 years of experience following local standards of care. Radiation dose was assessed in both study groups for initial diagnostic procedure. Details of the calculation method were described elsewhere [21].

### CT image acquisition and reading

CTA was performed on one of two 320-row CT scanners (Aquilion ONE^TM^, Canon Medical Systems, in 121 patients and Aquilion ONE ViSION Edition in 44 patients). Two patients assigned to the CTA group underwent ICA based on the clinician’s decision, leaving 165 patients who underwent CTA. Standard soft tissue and lung reconstructions of the raw data with 3–5-mm slice thickness on a large 320-mm FOV with the center of the reconstruction window at 75–80% of the RR interval were generated for assessment of noncardiac structures. All CT images were independently reviewed for ECFs by two readers, at least one of them a board-certified radiologist with a minimum of 5 years of experience. The final decisions were made by consensus. Clinically relevant ECFs were reported to the patient’s clinical physician, followed by immediate further management if required.

### Assessment of ECFs

An ECF was defined as an abnormality that was detected on CTA or ICA scans and located outside the heart and pericardium. First, we distinguished clinically relevant and nonsignificant ECFs. Categorization of ECFs was largely based on the classification proposed by Karius et al [24]. Clinically relevant ECFs were defined as requiring immediate therapy and/or further workup including additional imaging [24]. All other ECFs were classified as clinically nonsignificant. In the set of clinically relevant ECFs, we further tried to identify acutely life-threatening and malignant ECFs. Second, we quantified the diagnostic actions recommended for ECFs and analyzed whether they were followed by patients and if they had any therapeutic consequences. A therapeutic consequence was assumed if the patient received particular treatment for a clinically relevant ECF. Third, we evaluated if ECFs could potentially explain atypical chest pain. Based on theoretical considerations, we sought for alternative conditions that could account for a patient’s symptoms such as pneumonia, hiatal hernia, or cancer. All ECFs were assigned to one of five anatomical categories (lungs, upper abdomen, bones, vessels, and mediastinum) for quantitative analysis of their anatomical sites and distribution. The remaining ECFs were assigned to the category of “other adjacent regions”. Upon request of the editor in chief, we supplemented this prespecified analysis by an analysis of the potential of ECFs to explain the patient’s chest pain. Furthermore, we analyzed a potential association between the number of detected ECFs and patient age, sex, and smoking behavior defined as prior or current smoking.

### Follow-up of ECFs

Long-term follow-up for a median of 3.75 years was available. Follow-up data were available from follow-up questionnaires completed by the study participants. The patients were contacted by phone if some responses were missing or unclear. Additional follow-up data were available from the patients’ electronic medical record.

### Statistical analysis

All statistical analyses were performed with SAS University Edition 9.4 software. Categorical variables are presented as percentage (%) and continuous normally distributed variables as mean and standard deviation, while not normally distributed continuous outcomes are presented as median and interquartile range. We used Fisher’s exact test to compare presence of the ECFs between the two groups. Both outcomes (clinically relevant and nonsignificant) met the Poisson distribution (*p* = 0.838, *p* = 0.32, respectively); nevertheless, variance was larger than the mean for both outcomes. Consequently, univariate analysis of the number of detected ECFs and each independent variable (patient age, sex, and smoking behavior) was performed by negative binomial regression. A *p*-value < 0.025 was considered to indicate a statistically significant difference in multivariate negative binomial regression analysis.

## Results

### Detection of relevant ECFs

**Fig. 1:**
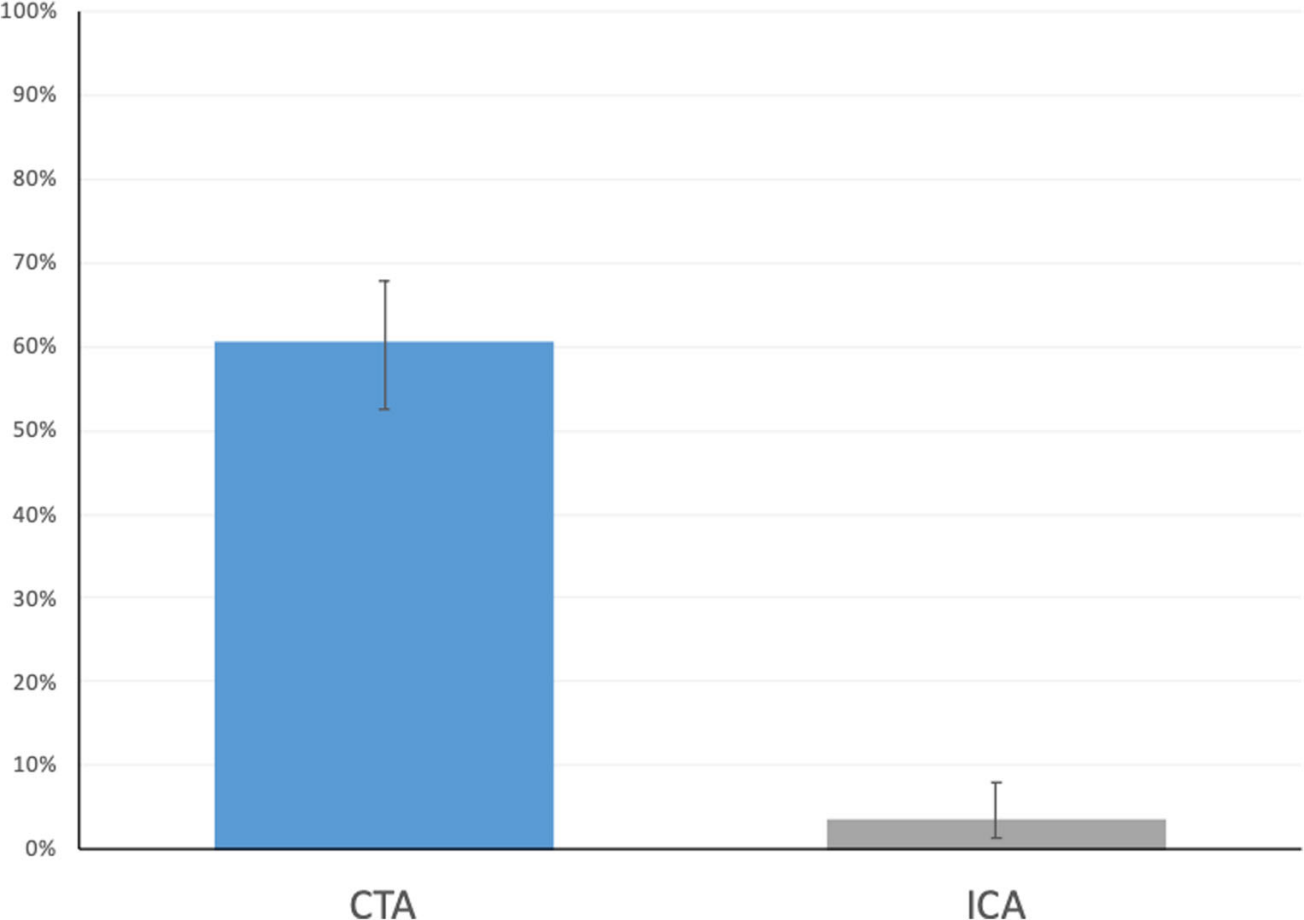
Prevalence of incidental ECFs in the CTA and ICA groups of our study

**Fig. 2:**
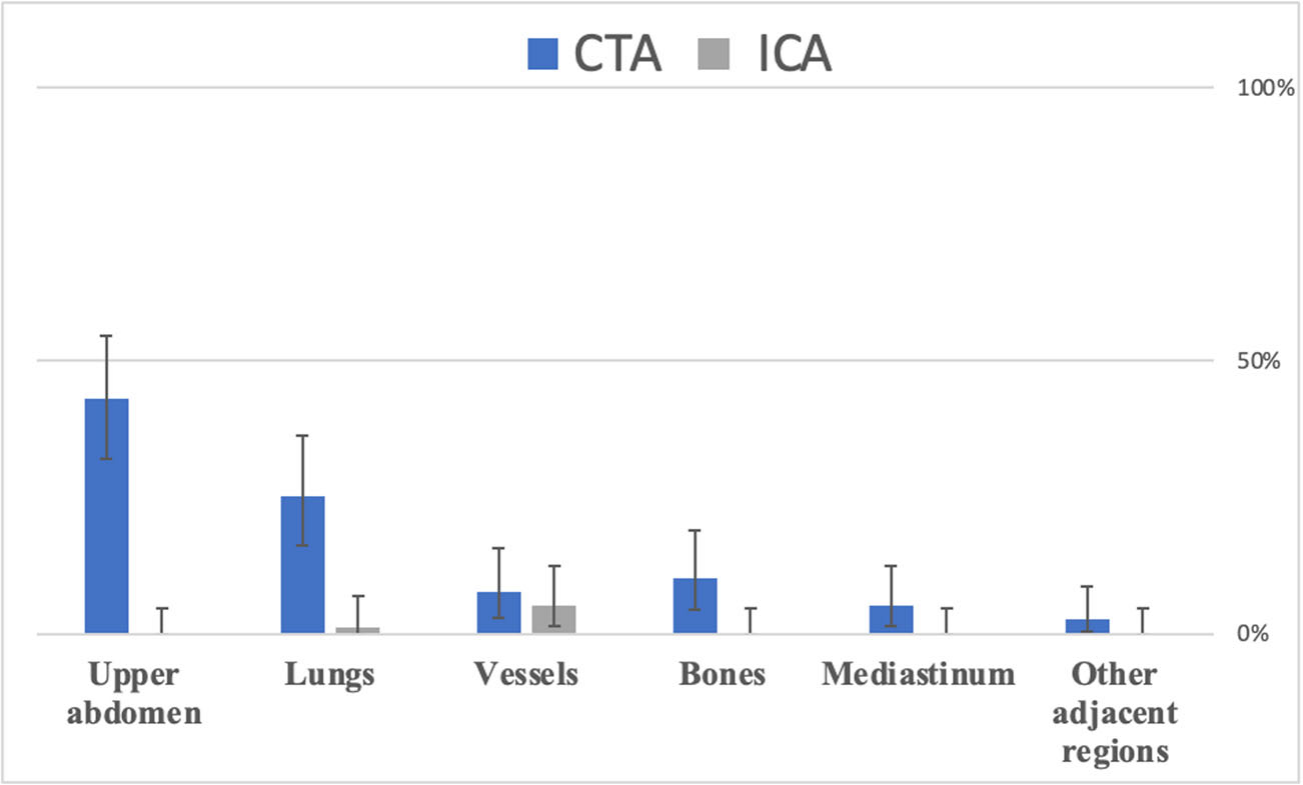
Distribution of clinically relevant ECFs by anatomical region

Baseline patient characteristics in the CTA and ICA groups are presented in ESM Table 1. One of 168 patients in the CTA group and 10 of 172 patients in the ICA group withdrew informed consent and did not undergo the assigned procedure. Consequently, 329 patients were available for analysis. The median exposure to radiation was 4.8 mSv (interquartile range: 4.1–5.8) in the CTA group and 6.0 mSv in the ICA group (3.0–10.0). Seventy-nine clinically relevant ECFs were detected in 59 patients (59/329, 17.9%; CTA: 55/167, 32.9%; ICA: 4/162, 2.5%). In 43 patients (13.1%; CTA: 40, ICA: 3), one clinically relevant ECF was present, whereas 16 patients (4.9%; CTA: 15, ICA: 1) had two or more clinically relevant ECFs. Overall, ECFs were found in 107 of 329 patients (32.5%), among them 101 in the CTA and only six in the ICA group (CTA: 101/167, 60.5%; ICA: 6/162, 3.7%; *p* < .001) (Fig. 1). These 107 patients had a total of 186 ECFs. Details of the distribution and frequencies of ECFs by group (CTA versus ICA) and anatomy are given in ESM Table 2. The distribution of clinically relevant ECFs by anatomical region is shown in Fig. 2. The most common ECFs were hiatal hernia (30.3%), suspicious pulmonary nodules (13.9%), and liver abnormalities (11.4%).

### ECFs as a possible cause of chest pain

**Fig. 3:**
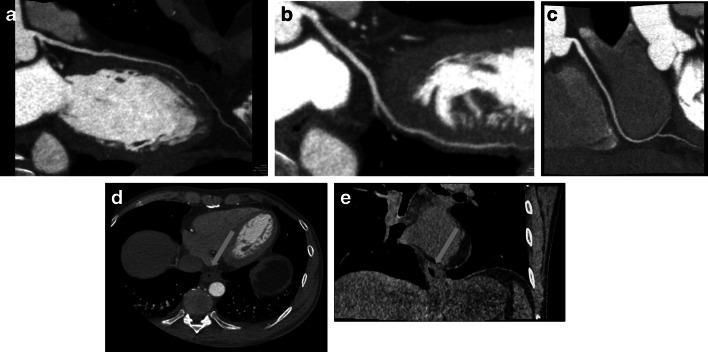
Forty-eight-year-old man with a 3.7 × 4.0 cm esophageal hiatal hernia (*arrow*), which after gastroenterological consultation and initiation of acid blocker treatment turned out to be a potential cause of chest pain in this patient. CTA detected no significant coronary artery stenoses in this patient. **a** LMA and LAD, curved reconstruction. **b** LCX, curved reconstruction. **c** RCA curved reconstruction. **d** Soft tissue reconstruction, axial plane. **e** Soft tissue reconstruction, coronal plane

Among the 329 patients available for analysis, 144 had atypical angina, 177 nonanginal chest pain, and 8 had other chest discomfort. In the CTA group, we detected ECFs that could potentially explain atypical chest pain in 13 of 101 patients with ECFs (12.9%). Moreover, in all 13 of those patients, CTA ruled out significant CAD (coronary stenosis > 50%), precluding the need for ICA. Thus, the potential risk associated to ICA might have been avoided and dedicated therapy initiated instead. That is a definite benefit for patients undergoing CTA. Conversely, in the ICA group, none of the ECFs could explain chest pain. All ECFs that required further imaging, consultation, or therapy were immediately reported to the patient and patient’s clinical physician. All recommendations were followed. Hiatal hernia (Fig. 3) was the most common ECF potentially causing chest pain (Table 1). At the time of the last follow-up performed after initiation of treatment in the 13 patients with ECFs potentially explaining symptoms, chest pain was improved in four patients (4.0% of those with ECFs on CTA) and resolved in seven patients (6.9%), leaving only two patients who still had chest pain. We did not detect any acutely life-threatening ECFs such as pulmonary embolism in either the CTA or the ICA group. Conversely, five malignant ECFs were detected in three patients (3/59; 5.1%), all of them in the CTA group. Malignancies were most commonly located in the lung, followed by the mediastinum and liver (Table 2).

**Table 1:** ECFs potentially explaining chest pain

ECFs potentially causing chest pain	Absolute number	Study group		Diagnosis of significant CAD		Prevalence
		CTA	ICA	Yes	No	
Upper abdomen						
Hiatal hernia	7	7	0	0	7	2.1%
Bones						
Spinal degeneration	2	2	0	0	2	0.6%
Forestier disease	1	1	0	0	1	0.3%
Lungs						
Pneumonia	2	2	0	0	2	0.6%
Lung cancer	1	1	0	0	1	0.3%
No. of patients with ECFs potentially explaining chest pain	13	13	0	0	13	4.0%

There was only one extracardiac finding potentially leading to chest pain in each patient

*ECF* extracardiac finding, *CTA* coronary computed tomography angiography, *ICA* invasive coronary angiography

**Table 2:** Malignant incidental ECFs

Malignant ECFs	CTA	ICA
Newly diagnosed		
Lung cancer	1	0
Previously known		
Lung metastasis	2	0
Liver metastasis	1	0
Mediastinal metastasis	1	0
Total no. of malignant ECFs	5	0
Total no. of patients with malignant ECFs	4	0
Frequency in relation to all clinically relevant ECFs in respective group	6.8%	0%
Frequency in relation to patients with clinically relevant ECFs in respective group	5.5%	0%

*ECF* extracardiac finding, *CTA* coronary computed tomography angiography, *ICA* invasive coronary angiography

### Recommendations and management of ECFs

Further imaging for workup of detected ECFs was recommended in 33 patients (10.0%; CTA: 30/167, 18.0%; ICA: 3/162, 1.9%). The most frequent follow-up imaging test was chest CT followed by abdominal ultrasonography (Table 3). Additional clinical consultations by a specialist were recommended in 26 patients (7.9%; CTA: 25/167, 15.0%; ICA: 1/162, 0.6%). The most frequently recommended specialty consultation was a gastrointestinal consultation followed by an orthopedic consultation (Table 3). Thus, a total of 72 diagnostic procedures (38 further imaging examinations, 34 clinical consultations) were recommended in 59 patients (CTA: 55; ICA: 4). Most patients followed the recommendations given based on their ECFs (49/59; 83.1%). In the CTA group, 10 patients (10/59; 16.9%) did not undergo the recommended diagnostic procedures. None of the patients in our study had adverse events related to the recommended additional test procedures or treatment.

**Table 3:** Frequency of recommended follow-up imaging investigations and clinic consultations based on detected ECFs on CTA vs. ICA

Follow-up imaging	Frequency		Follow-up clinic consultation	Frequency	
	CTA	ICA		CTA	ICA
Chest CT	17	1	Gastrointestinal	23	0
Abdominal ultrasonography	8	0	Orthopedic	7	0
Echocardiography	4	2	Pulmonary	3	1
Mammography	2	0			
Cardiac MRI	1	0			
PET/CT	1	0			
Chest plain radiography	1	0			
Thoracic spine MRI	1	0			
Total number of procedures	35	3		33	1

*ECF* extracardiac finding, *CTA* coronary computed tomography angiography, *ICA* invasive coronary angiography

**Fig. 4:**
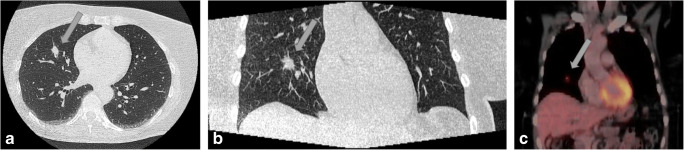
Incidentally detected poorly differentiated acinar adenocarcinoma of the lung in a 61-year-old woman with atypical chest pain. **a** Spiculated 9 × 14 mm consolidation in segment 5 of the right lung in CTA (*arrow*), axial plane. **b** CTA, coronal plane. **c** Subsequent PET-CT examination confirms lung cancer (*arrow*)

Patients received appropriate therapy based on detected ECFs if required. In 17 patients, the clinically relevant ECFs had therapeutic consequences (17/329, 5.2%; CTA group: 16/167, 9.6%, ICA group: 1/162, 0.6%) (Table 4). In 12 patients in the CTA group (12/17; 70.6%), the therapeutic consequence was medical therapy (antacid medication in 9 patients, antibiotics in 2 patients, and antihypertensive in 1 patient). Two patients each in the CTA group (2/17; 11.8%) underwent physical therapy and surgery (thoracotomy for lung cancer and surgery of upside-down stomach). The case of newly diagnosed lung cancer is presented in Fig. 4. This patient had curative thoracic surgery and chemoradiotherapy and has since been free of recurrence for almost 5 years. The one patient with therapeutic consequences for ECFs in the ICA group was newly diagnosed with pulmonary hypertension. As a result of this ECF, the planned aortic valve replacement therapy had to be canceled. Table 4 summarizes clinically relevant ECFs by anatomical territory and in relation to randomized group and gives information if ECFs had therapeutic consequences.

**Table 4:** Clinically relevant incidental ECFs by anatomical region and therapeutic consequences.

ECFs by anatomical region	Coronary computed tomography angiography (CTA)		Invasive coronary angiography (ICA)	
	Therapeutic consequences			
	No (*n* = 58)	Yes (*n* = 16)	No (*n* = 4)	Yes (*n* = 1)
Upper abdomen (*n* = 34)				
Hiatal hernia	14	10		
Liver hemangioma/mass/cystic lesion	8			
Malignancy	1			
Adrenal mass	1			
Lungs (*n* = 21)				
Suspicious pulmonary nodules	11			
Malignancy	2	1		
Chronic changes of lung parenchyma and bronchial system	2			
Pulmonary infiltration		2		
Pulmonary hypertension		1		1
Pleural effusion	1			
Vessels (*n* = 10)				
Aortic aneurysm	2		1	
Aortic stenosis			2	
Dilatation of pulmonary arteries	1			
Other abnormalities of aorta	3		1	
Bones (*n* = 8)				
Spinal degeneration/destruction	4	2		
Forestier disease	2			
Mediastinum (*n* = 4)				
Enlarged lymph node	1			
Mediastinal mass	1			
Mediastinal malignancy	1			
Thymus hyperplasia	1			
Other adjacent regions *(n* = 2)				
Breast lesion	2			

Therapeutic consequences were assumed to be present if the patient received particular treatment aimed at the clinically relevant ECF

*ECF* extracardiac finding, *CTA* coronary computed tomography angiography, *ICA* invasive coronary angiography

### Impact of variables on prevalence of ECFs

We analyzed the influence of patient age, sex, and smoking behavior on the prevalence of ECFs, separately for all, clinically relevant, and clinically nonsignificant ECFs. The results of multivariate analysis of potential associations between any of the three variables and category of ECF are presented in Table 5. A statistically significant positive association was revealed between female sex and all ECFs as well as clinically relevant ECFs. Women were more likely to have clinically relevant ECFs than men (OR, 1.65; 95% CI 1.08–2.51; *p* = 0.019). For patient age, there was no significant correlation between increasing age and prevalence of ECFs. The same influence was observed regarding smoking behavior.

**Table 5:** Associations between risk variables and the presence of incidental ECFs

Risk variable	All ECFs		Clinically nonsignificant ECFs		Clinically relevant ECFs	
	Odds ratio (CI)	*p* value	Odds ratio (CI)	*p* value	Odds ratio (CI)	*p* value
Female gender	1.38 (1.007–1.886)	0.045	1.08 (0.673–1.742)	0.74	1.652 (1.08–2.512)	0.019
Male gender	0.725 (0.530–0.993)	0.045	0.923 (0.574–1.484)	0.74	0.605 (0.398–0.920)	0.019
Smoking	1.202 (0.887–1.628)	0.235	1.353 (0.847–2.162)	0.206	1.103 (0.741–1.644)	0.629
Age > 57 years	1.196 (0.863–1.659)	0.282	1.469 (0.877–2.462)	0.144	1.035 (0.678–1.580)	0.874

*CI* confidence interval, *ECF* extracardiac finding

## Discussion

In this study, we performed systematic long-term follow-up for a median of 3.75 years of initially suspicious ECFs found on CTA and ICA in patients with atypical chest pain and suspected CAD who participated in our randomized trial. Thus, we could evaluate the potential benefit of detection of relevant ECFs and analyze the consequences of the different detection rates of ECFs on CTA vs. ICA. Our study shows that the higher detection rate of ECFs by CTA is clinically beneficial as the findings include potential differential diagnoses that explain chest pain in patients in whom the diagnostic procedure rules out significant CAD. This could eliminate the need to perform ICA in such cases, which is invasive and associated with more procedural complications than CTA. Finally, detection of relevant ECFs results in more diagnostic procedures. While this may be associated with potential procedural complications and increase healthcare costs, it can improve quality of life of affected patients.

**Fig. 5:**
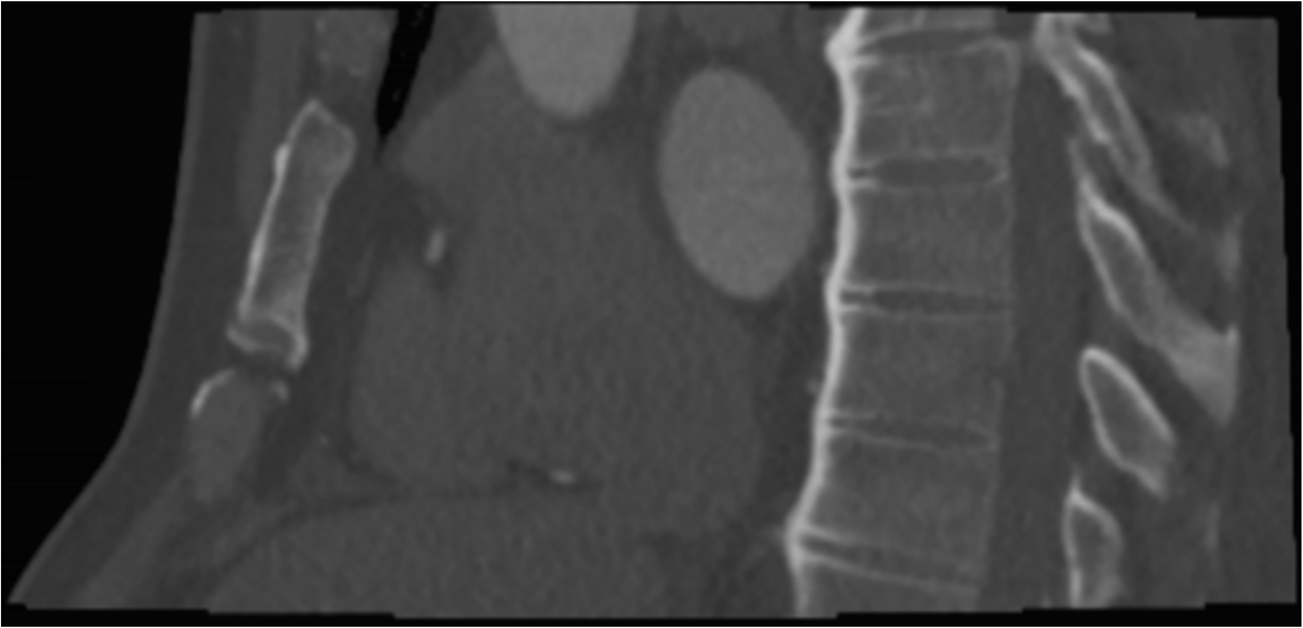
Fifty-six-year-old man with atypical load-independent chest pain not extending further. Diffuse idiopathic skeletal hyperostosis (DISH), also referred to as Forestier disease, was diagnosed and treated with analgesics and physical therapy, which contributed to chest pain relief

Two studies performed so far reported clinically relevant ECFs on CTA as a possible underlying cause of chest pain when CAD was ruled out by the procedure [1, 2]. Karius et al [1] investigated 2330 patients with chest pain, identifying 7.9% ECFs that might explain their pain. In a study population of 1778 patients, Williams et al [2] identified ECFs that were then assessed as possible alternative causes of chest pain in 3% of cases. In our study, 4.0% of patients were diagnosed with ECFs on CTA which could cause anginal symptoms. Following initiation of treatment in this subgroup, chest pain was improved in 4% and resolved in 7% of all patients in whom ECFs were detected by CTA. The most common findings were hiatal hernia (Fig. 3) and spinal abnormalities such as Forestier disease (Fig. 5), similar to the study of Karius et al. The use of large FOV resulted in higher detection of ECFs [7]. The novelty of our study is that we performed a long-term analysis of clinical consequences of relevant ECFs in patients with atypical chest pain and suspected CAD randomly assigned to CTA or ICA. We systematically followed up detected ECFs over 3 years to analyze the potential clinical benefit of their detection for patients.

Obviously, the detection of ECFs leads to more diagnostic procedure with potential complications, especially when an invasive procedure is needed, such as a lung biopsy. However, the vast majority of recommended follow-up procedures were noninvasive diagnostic imaging tests such as chest CT and abdominal ultrasonography. Nonetheless, in our study, none of the patients had adverse events related to the recommended diagnostic procedures. Neither Williams et al nor Bendix et al, who both investigated patients with chest pain, reported any adverse events related to follow-up procedures [2, 3]. It is also clear that more diagnostic procedures mean extra healthcare costs, but as stated before, diagnostic workup of ECFs can improve quality of life, which is a definite advantage for affected patients. Thus, our results seem to underline the fundamental importance of including a systematic analysis of ECFs in order to identify other causes that could potentially explain chest pain.

Furthermore, ours is the first study that found a significant positive association of female sex with clinically relevant ECFs. Our results suggest that women are more likely to have clinically relevant ECFs than men. A possible explanation for this observation is that women may have a lower pretest probability of CAD than men and consequently a higher probability of suffering from chest pain due to other, noncardiac causes [25]. An earlier study suggests that women with atypical chest pain and a clinical indication for ICA benefit from a reduction of minor procedural complications when undergoing CTA instead of ICA [26]. Our study suggests that the detection of ECFs which may potentially explain atypical chest pain is another clinical benefit for women.

It is already known that, in patients with atypical angina and a low to intermediate risk of CAD, performing CTA first defers ICA with no increase in long-term events, reduces minor procedural complications, and shortens the hospital stay compared to direct coronary angiography [21]. Our study has revealed an additional benefit of performing CTA in patients with atypical symptoms, namely the detection of ECFs which might constitute alternative causes of chest pain. Such incidental findings may contribute to the initiation of efficient diagnostic workup and therapy, thus eliminating the source of chest pain or even curing a potentially fatal malignancy.

## Limitations

Our study has several important limitations. First, due to a lack of other options, we used our subjective theoretical considerations to define the type and prevalence of ECFs classified as potentially explaining chest pain. Some follow-up examinations performed on an outpatient basis might not have been included if not consistently reported in the patient’s follow-up questionnaire. Finally, the single-center design and the rather small number of patients included are important limitations. To obtain robust data on the clinical effectiveness and transferability to different clinical settings, investigation of a larger patient population and with longer follow-up and ideally in a multicenter trial is recommended. All of these requirements might be fulfilled by our ongoing multicenter randomized controlled DISCHARGE trial [27].

## Conclusions

Our study is the first randomized comparison and shows that patients presenting with atypical angina or chest pain and a low to intermediate risk of CAD may have a twofold benefit from undergoing CTA instead of ICA: (1) detection of ECFs allowing early initiation of treatment or as potential explanation of patients’ symptoms and (2) exclusion of obstructive CAD. Actionable clinically relevant ECFs detected by CTA affect patient management and therapy and may thus improve chest pain and consequently quality of life. Most of these ECFs would not have been detected by ICA.

## Supplementary information

ESM 1 Table 1: Baseline patient characteristics assigned to CTA or ICA. Table 2: Spectrum of ECFs (DOCX 27 kb)

## Abbreviations

CAD: Coronary artery disease
CI: Confidence interval
CTA: Coronary computed tomography angiography
ECF: Extracardiac finding
FOV: Field of view
ICA: Invasive coronary angiography

## Declarations

### Guarantor

The scientific guarantor of this publication is Prof. Dr. Marc Dewey

### Statistics and biometry

One of the authors has significant statistical expertise.

### Methodology

• retrospective

• randomized controlled trial

• performed at one institution
