# Supplementary material for: Detection of relevant extracardiac findings on coronary computed tomography angiography vs. invasive coronary angiography
Source: Eur Radiol. 2021 Jun 15;32(1):122–31. doi: 10.1007/s00330-021-07967-x (PMC8660731; doi:10.1007/s00330-021-07967-x)
Supplement: Supplementary file 1 — Table 1: Baseline patient characteristics assigned to CTA or ICA. Table 2: Spectrum of ECFs (DOCX 27 kb) [file 330_2021_7967_MOESM1_ESM.docx]

**ESM Table 1** Baseline patient characteristics assigned to CTA or ICA.

| **Characteristics** | Coronary computed tomography angiography (CTA)  n=167 | Invasive coronary angiography (ICA)  n=162 |
| --- | --- | --- |
| No. of women (%)  Age (years)  Cardiovascular risk factors:  No. with diabetes mellitus (%)  No. with arterial hypertension (%)  No. with hyperlipidemia (%)  No. of current smokers (%)  No. of former smokers (%)  Clinical presentation:  Atypical angina (%)  Nonanginal chest pain (%)  Other chest discomfort (%) | 88 (52.7)  60.4 ± 11.3  15 (9.0)  111 (66.5)  95 (56.9)  41 (24.5)  47 (28.1)  65 (38.9)  97 (58.1)  5 (3.0) | 78 (48.1)  60.4 ± 11.4  30 (18.5)  112 (69.1)  81 (51.0)  34 (21.0)  51 (31.5)  79 (48.8)  80 (49.4)  3 (1.8) |

If not otherwise stated, numbers presented are absolute numbers with percentages in brackets.

CTA: coronary computed tomography angiography

ICA: invasive coronary angiography

**ESM Table 2**: Spectrum of ECFs

| **ECFs** | | **Number of ECFs** | **Relative frequency** | **Cumulative frequency** | **CTA** | **ICA** |
| --- | --- | --- | --- | --- | --- | --- |
| **Lungs** | | **73** | **39.25%** | **39%** | **72** | **1** |
| Pulmonary nodules | | 37 | 50.68% | 51% | 37 | 0 |
|  | Unsuspicious pulmonary nodule | 26 | 35.62% | - | 26 | 0 |
|  | Suspicious pulmonary nodule | 11 | 15.07% | - | 11 | 0 |
| Chronic changes of lung parenchyma and bronchial system | | 18 | 24.66% | 76% | 18 | 0 |
| Atelectasis/dystelectasis | | 9 | 12.33% | 88% | 9 | 0 |
| Malignancy | | 3 | 4.11% | 92% | 3 | 0 |
| Pulmonary hypertension | | 2 | 2.74% | 95% | 1 | 1 |
| Pulmonary infiltration | | 2 | 2.74% | 98% | 0 | 0 |
| Abnormalities of pleura | | 2 | 2.74% | 100% | 2 | 0 |
|  | Pleural effusion | 1 | 1.37% | - | 1 | 0 |
|  | Pleuritis | 1 | 1.37% | - | 1 | 0 |
|  | | | | | | |
| **Upper abdomen** | | **51** | **27.42%** | **67%** | **51** | **0** |
| Hiatal hernia | | 41 | 80.39% | 80% | 41 | 0 |
| Liver abnormalities | | 9 | 17.64% | 98% | 10 | 0 |
|  | Haemangioma/mass/cystic lesion | 8 | 15.69% | - | 9 | 0 |
|  | Malignancy | 1 | 1.96% | - | 1 | 0 |
| Kidney abnormalities | | 1 | 1.96% | 100% | 1 | 0 |
|  | Adrenal mass | 1 | 1.96% | - | 1 | 0 |
|  | | | | | | |
| **Bones** | | **24** | **12.90%** | **80%** | **24** | **0** |
| Abnormalities of the spine | | 23 | 95.83% | 96% | 23 | 0 |
|  | Degeneration/destruction | 17 | 70.83% | - | 17 | 0 |
|  | Haemangioma | 3 | 12.50% | - | 3 | 0 |
|  | Forestier disease | 2 | 8.33% | - | 2 | 0 |
|  | Scoliosis | 1 | 4.17% | - | 1 | 0 |
| Pectus anomaly | | 1 | 4.17% | 100% | 1 | 0 |
|  | | | | | | |
| **Vessels** | | **24** | **12.90%** | **92%** | **17** | **6** |
| Aortic abnormalities | | 16 | 66.67% | 67% | 9 | 6 |
|  | Stenosis | 3 | 12.50% | - | 0 | 3 |
|  | Elongation | 3 | 12.50% | - | 2 | 1 |
|  | Aneurysm | 3 | 12.50% | - | 2 | 1 |
|  | Dilatation/ectasia | 3 | 12.50% | - | 2 | 0 |
|  | Other abnormalities of aorta | 4 | 16.67% | - | 3 | 1 |
| Atherosclerosis | | 7 | 29.17% | 96% | 7 | 0 |
| Abnormalities of pulmonary arteries | | 1 | 4.17% | 100% | 1 | 0 |
|  | Dilatation of pulmonary arteries | 1 | 4.17% | - | 1 | 0 |
|  | | | | | | |
| **Mediastinum** | | **11** | **5.91%** | **98%** | **11** | **0** |
| Enlarged/calcified lymph node | | 8 | 72.73% | 73% | 8 | 0 |
| Thymus abnormalities | | 1 | 9.09% | 82% | 1 | 0 |
|  | Thymus hyperplasia | 1 | 9.09% | - | 1 | 0 |
| Mediastinal mass | | 1 | 9.09% | 91% | 1 | 0 |
| Mediastinal malignancy | | 1 | 9.09% | 100% | 1 | 0 |
|  | | | | | | |
| **Other adjacent regions** | | **3** | **1.61%** | **100%** | **3** | **0** |
| Breast abnormalities | | 3 | 100.0% | 100% | 3 | 0 |
|  | Lesion | 2 | 66.67% | - | 2 | 0 |
|  | Calcification | 1 | 33.33% | - | 1 | 0 |
|  | | | | | **CT** | **ICA** |
| **Total number of ECFs** | | **186** |  | | **179** | **7** |
| **Total number of patients in study cohort** | | **329** |  | | **167** | **162** |
| **Total number of patients with any ECF** | | **107** |  | | **101** | **6** |
| **Prevalence of any ECF** | | **32.52%** |  | | **60.48%** | **3.70%** |

ECF: extracardiac finding

CTA: coronary computed tomography angiography

ICA: invasive coronary angiography
